# The digital readiness of future physicians: nurturing the post-pandemic medical education

**DOI:** 10.1186/s12913-024-11365-6

**Published:** 2024-08-02

**Authors:** Marta Marsilio, Valeria Calcaterra, Gabriele Infante, Martina Pisarra, Gianvincenzo Zuccotti

**Affiliations:** 1 Department of Economics, Management and Quantitative Methods (DEMM), University of Milan, Milan, 20122 Italy; 2 Pediatric and Adolescent Unit, Department of Internal Medicine, University of Pavia, Pavia, 27100 Italy; 3 Pediatric Department, Buzzi Children’s Hospital, Milan, 20154 Italy; 4 Department of Biomedical and Clinical Sciences, University of Milan, Via GB Battista n. 74, Milano, 20157 Italy

**Keywords:** Medical education, Telemedicine, Digital health, Digital readiness, Health literacy

## Abstract

**Background:**

The COVID-19 pandemic has accelerated the integration of digital technologies in the healthcare sector. Telemedicine has notably emerged as a significant tool, offering a range of benefits. However, various barriers, such as healthcare professionals’ insufficient technological skills and competencies, can hinder its effective implementation. Scholars have examined the readiness of future physicians, with some studies exploring their readiness before or during the COVID-19 crisis. There is, however, a noteable gap in the literature concerning the post-pandemic period. This study aims to identify gaps in current medical education programs by examining two primary aspects: (1) technical readiness (encompassing general and health-related digital competencies) and (2) behavioural readiness, which includes prior experiences and future intentions related to telemedicine education and implementation among medical students and residents.

**Methods:**

A cross-sectional study was conducted using a web-based questionnaire administered to medical students and residents at a major Northern Italian university. The survey responses were analyzed to ascertain whether their distributions varied across demographic variables such as gender and level of education.

**Results:**

The most commonly owned technologies were laptops and smartphones, with smartphones perceived as the easiest to use, while desktop computers presented more challenges. Approximately 38% of respondents expressed apprehension about applying digital health information in decision-making processes. There was a significant lack of both personal and academic experience, with only 16% of students and residents having used telemedicine in a university setting. Despite this, 83% of participants expressed a desire for training in telemedicine, and 81% were open to experimenting with it during their academic journey. Moreover, 76% of respondents expressed interest in incorporating telemedicine into their future clinical practice.

**Conclusions:**

This study highlights the need for medical students and residents to receive specific education in digital health and telemedicine. Introducing curricula and courses in this domain is critical to addressing the challenges of digital healthcare.

**Supplementary Information:**

The online version contains supplementary material available at 10.1186/s12913-024-11365-6.

## Background

Telemedicine has provided numerous benefits to the reorganization of healthcare services, including enhanced accessibility, mitigation of personnel shortages, reduction in hospitalizations for chronic diseases, and minimization of service waiting lists [1–3]. However, realising these benefits requires considering various facilitators and potential barriers [4–6]. An essential aspect of this discourse is the pivotal role that medical education plays in preparing future physicians to effectively integrate digital technologies, such as telemedicine applications, into their clinical practice [7]. This requires the development of technological skills and competencies, as well as hands-on experience with telemedicine, for both medical students and residents throughout their educational pathways. With the rapid expansion of telemedicine in clinical practice, integrating telemedicine into medical education programmes becomes a priority for policy makers. The literature increasingly highlights deficiencies in technological knowledge and skills among medical students and residents, as well as their lack of experience with telemedicine [8–11]. Studies show that while medical students and residents are eager to be involved in telemedicine training and practice, a gap in existing curricula is evident. Some research offers guidance for developing telemedicine educational interventions by demonstrating improvements in students’ competencies [12]. A systematic review of telemedicine curricula within undergraduate programs, mostly conducted in the USA, indicates high student satisfaction despite the small sample sizes and exploratory nature of these studies, which primarily examined the pre- and during Covid-19 sitautions. Moreover, while for example some students’ gender gaps exist with ICT fields, evidence exploring differences based on demographic information is lacking [13].

COVID-19 marked a turning-point in the healthcare sector, prompting significant investments [14] to fortify the digital transformation of national health systems in the post-pandemic era [15–17]. Among the various areas of digital innovation in healthcare, the field of telemedicine became central to patient healthcare delivery during the COVID-19 pandemic era’ [18, p.583] enabling safe implementation of pandemic control measures [19] and demonstrating strong evidence of visible outcomes on a global scale [20]. Therefore, telemedicine has been recommended as a valid alternative to the traditional provisional model of healthcare services [18]. Although telemedicine grew exponentially during the COVID-19 pandemic which acted as a catalyst, it is still expected to grow annually by 18.9% from 2020 to 2030 [21], highlighting the growing importance of this digital technology for both public managers and policy makers.

However, a noticeable gap still exists in the literature concerning the post-pandemic situation of the next generation physicians in terms of digital preparedness, as well as of their experience and willingness to participate in telemedicine programmes.

To address this gap, this study aims to investigate the digital readiness of medical students and residents, providing insights for designing post-pandemic medical education programs.

To achieve this goal, a cross-sectional study was conducted, examining both the (1) technical readiness, which includes the general and health-related digital competencies of medical students and residents, assessed through scientific and validated scales; and (2) behavioural readiness, investigating their previous personal and professional (e.g., internships) experiences and future intentions related to telemedicine education and implementation. Demographic variables such as gender and level of education were also considered in this analysis. The study’s findings offer critical insights into the adequacy of post-COVID medical education programs and underscore the need for potential restructuring to keep pace with advancements driven by the proliferation of digital technologies in the healthcare sector.

## Methods

### Study design and setting

The study employed an observational cross-sectional design, adhering to the consensus-based checklist for reporting survey studies [22]. The web-based survey, conducted anonymously and voluntarily from February to April 2023, underwent a pilot sample evaluation to identify any necessary amendments or clarifications. The final survey was administered to medical students and residents across various medical fields enrolled in the academic year 2022/2023 at the University of Milan, a prominent institution in Northern Italy.

Italy was selected as the study setting due to its significant investment in telemedicine. Italian public healthcare organizations are allocating substantial funds through the National Recovery and Resilience Plan (NRRP) funded by the NGEU initiative to recover from the COVID-19 pandemic. Specifically, 1 billion Euros have been allocated for telemedicine to provide care for at least 200,000 patients by 2025. Furthermore, the University of Milan has been actively involved in the provision of telemedicine initiatives during COVID-19 pandemic [23] and it has recently planned to establish a University Telehealth Hub to cultivate telemedicine knowledge and skills through simulations and training programs.

At the time of the study, the university had not yet implemented any formal telemedicine curriculum or structured course. Integration of references and excursions into the use of telemedicine in medical practice was left to the discretion of each professor across various conventional courses within the medical program. The survey was distributed through the university’s institutional email system, employing a quota sampling design [24] to ensure a sufficient number of participants for statistical evaluation. The study adhered to the guidelines of the Declaration of Helsinki and received approval from the Institutional Review Board at the University of Milan (protocol number 21/23). Data collection was conducted using the EUSurvey web platform developed by the European Commission’s Directorate-General for Informatics (https://ec.europa.eu/eusurvey/).

### Questionnaire

The online questionnaire developed for this study (Additional File 1), comprising 25 questions, was divided into three sections: Demographical, Technical, and Behavioural. Demographic information included gender, age, level of education, and the year of study (for medical students) or year of specialization (for resident physicians). Drawing from previous research [25–27], the *technical* section investigated the digital competences of medical students and residents, encompassing technological skills and knowledge, as well as digital and e-health literacy, while the *behavioural* investigated participants’ experiences, attitudes, and behavioural intentions with telemedicine, including prior experiences, desire for training and education, and intentions to use telemedicine.

#### Technical component

This component comprised the following two sections.

The *digital technology knowledge and skills* section explores attitudes and usage frequency of Information and Communication Technology (ICT) devices (e.g., smartphone, tablet, PC) and assesses possession of basic digital skills. The evaluation of basic digital skills follows the proficiency in five areas of digital competence outlined in the self-assessment grid proposed by the DigComp framework [28, 29] and the novel DigComp 2.2 framework [30]. Further questions have been added to investigate: participants’ reactions when learning to use new computer or smartphone applications, the types of information and communication technology (ICT) devices they own at home, their self-assessment of computer and digital device usage proficiency, frequency of technology use in their daily routines, internet access and wireless connection at home, as well as their possession of various digital skills in information retrieval, communication, content creation, security measures, and problem-solving abilities.

The *digital health knowledge and skills* section first evaluates e-health literacy using the eight-point Likert scale of the Italian Version of the e-health Literacy Scale (IT-eHEALS) [31], based on Norman and Skinner (2006) [32]. Validation on a student population by Bravo et al. (2018) demonstrated solid internal consistency (Cronbach’s alpha 0.90) ) [31]. A total score as sum of single items is justified by a factor variance equal to 61%. Two additional items on COVID-19, drawn from previous studies, were added to assess the potential impact of the pandemic [33]. This section also explores the presence of health-related applications on participants’ smartphones, indicating mHealth adoption, and the use of technology for various health-related activities (e.g., reading medical articles, checking test results, scheduling appointments) to quantify eHealth adoption. Additionally, the respondents’ level of information about telemedicine and their understanding of situations fitting the definition of telemedicine were investigated.

#### Behavioural component

Drawing from previous studies, this component first explores participants’ *previous experience with telemedicine* through four questions assessing their familiarity with telemedicine, involvement in telemedicine projects for themselves or others, participation in telemedicine projects during the academic pathway, and specific services utilized, exploring teleconsultation, telemonitoring, telecontrol, and telerehabilitation.

Secondly, it investigates participants’ *interest in telemedicine*, examining their attitudes and beliefs while also exploring their interest in telemedicine training and usage during their studies, as well as their interest in utilising telemedicine in their future practice.

Finally, the questionnaire focuses on *perceptions of advantages and disadvantages* of telemedicine for patients, as well as their views on challenges related to telemedicine and the potential benefits it could offer to healthcare professionals.

### Statistical data analysis

Quantitative variables were characterized using common measures of central tendency, while categorical variables were presented as counts and percentages. Likert scale variables were treated both as ordinal categorical and quantitative variables, as deemed appropriate.

For exploring associations and correlations, inferential non-parametric bilateral tests were employed, with a significance threshold (α) set at 0.05. Non-parametric tests have the advantage of exploring associations without assuming any specific parametric distribution, relying instead on ranks or exact calculations. Unlike parametric tests, such as the t-test and chi-square test, non-parametric tests are particularly suitable when small sample sizes or many subgroups are involved, ensuring robust and reliable results. Specifically, the Wilcoxon-Mann-Whitney (WMW) test [34] was utilized to evaluate continuous variables against binary indicators to test if the two sub- distributions could be considered equal. The Fisher-Freeman-Halton (FFH) exact test [35] was applied for contingency tables to test the null hypothesis of independence between the two factors. For Likert scale variables, both tests were conducted, deeming an association present if both were statistically significant.

Concerning specific analyses, the survey responses were examined to ascertain whether their distributions varied across demographic variables such as gender (female, male, and non-binary) and level of education (medical students and resident physicians). Multiple-choice questions were also delineated in terms of item combination frequencies.

In the case of the IT-eHEALS scale, the eight constituent items underwent reliability analysis, reporting Crombach’s alpha, percentage of explained variance, and eigenvalue. Based on the reliability results, the overall scale score was calculated as the sum of individual items within a range from 0 to 32 and treated as a continuous variable. Two additional items on eHealth literacy were scored separately and scaled within the same range.

## Results

### Sample characteristics

The sample consists of 307 participants, with 140 (45.6%) identified as resident physicians and 167 (54.4%) as medical students. The power analysis, conducted as part of the study protocol, determined a minimum sample size of 306, based on the assumption of observing a similar eHEALS score distribution as reported in the Italian validation study on a student sample [31]. The response rate of 2.2% is attributed to the extensive number of addresses within the university’s database mailing list, encompassing individuals who are no longer attending the university.

Table 1 presents the characteristics of the participants, both overall and categorized by educational level.

**Table 1:** Participant demographics

Characteristics	Levels / Measures	Overall (*n* = 307)		Medical Students (*n* = 167)		Resident Physicians (*n* = 140)	
Gender, n (%)	Female	162	(52.8%)	90	(53.9%)	72	(51.4%)
	Male	142	(46.2%)	75	(44.9%)	67	(47.9%)
	Non-binary	3	(1.0%)	2	(1.2%)	1	(0.7%)
Age, years	Mean (SD)	26.5	(6.0)	23.4	(5.1)	30.1	(4.8)
	Median (IQR)	26	(22–29)	23	(20.5–24)	29	(27–31)
	Min - Max		(18–64)		(18–64)		(24–57)
Academic year, n (%)	1st	58	(28.9%)	29	(17.4%)	29	(20.7%)
	2nd	64	(20.9%)	23	(13.8%)	41	(29.3%)
	3rd	54	(17.6%)	24	(14.3%)	30	(21.4%)
	4th	60	(19.5%)	28	(16.8%)	32	(22.9%)
	5th	32	(10.4%)	24	(14.3%)	8	(5.7%)
	6th or higher	36	(12.7%)	36	(23.4%)		

### Digital readiness assessment

The results of the analysis of the two components of the digital readiness of medical students and residents are reported as follows. Disparities in item responses have not been parsed by age, as it is highly correlated with educational level (WMW *p* < .001), nor by non-binary gender subpopulation due to inadequate representation in the sample. As described in the Methods section, the WMW test was used to assess equality between two sub distributions of a numerical variable, while the FFH test was applied to test independency between two factors.

### Technical component

Table 2 summarises the results for the technical component, with gender and educational level symbols indicating statistically significant differences within each domain.

**Table 2:** Primary summary results of the survey technical component, overall and by educational level

Sections	Domains	Overall	Medical Students	Resident Physicians	
A. TECHNICAL COMPONENT					
Digital and technology knowledge and skills					
	Technology enthusiasm	227 (73.9)	123 (73.7)	104 (74.3)	
	Desktop				
	*Confident user*	180 (58.6)	93 (55.7)	87 (62.1)	§
	*Frequent user*	138 (45.0)	39 (23.4)	99 (70.7)	§†
	Laptop				
	*Confident user*	201 (65.5)	108 (64.7)	93 (66.4)	§
	*Frequent user*	270 (87.6)	146 (87.4)	123 (87.9)	
	Smartphone				
	*Confident user*	236 (76.9)	133 (79.6)	103 (73.6)	
	*Frequent user*	304 (99.0)	166 (99.4)	138 (98.6)	
	Tablet				
	*Confident user*	178 (58.0)	108 (64.7)	70 (50.0)	
	*Frequent user*	146 (47.6)	94 (56.3)	52 (37.1)	†
	Home Internet connection availability	298 (97.1)	163 (97.6)	135 (96.4)	
	Overall self-evaluated digital skill of use	262 (85.3)	143 (85.6)	119 (85.0)	§
	Digital skills proficiency by domain				
	*Information*	305 (99.3)	167 (100)	138 (98.6)	
	*Communication*	295 (96.1)	164 (98.2)	131 (93.6)	†
	*Content Creation*	278 (90.6)	146 (87.4)	132 (94.3)	†
	*Safety*	294 (95.8)	160 (95.8)	134 (95.7)	
	*Problem Solving*	287 (93.5)	154 (92.2)	133 (95.0)	
Digital health knowledge and skills					
	mHealth user	229 (74.6)	126 (75.4)	103 (73.6)	§
	eHealth user °	235 (76.5)	121 (72.5)	114 (81.4)	
	eHealth literacy (e-HEALS)	26.5 (SD 5.7)	25.3 (SD 6.0)	28.0 (SD 4.9)	†
	Covid-related eHealth literacy	24.0 (SD 6.1)	24.3 (SD 5.9)	23.6 (SD 6.2)	
	Telemedicine informed	133 (43.3)	55 (32.9)	78 (55.7)	†
	Telemedicine correct identification ^	224 (73.0)	115 (68.9)	109 (77.9)	

Notes: Absolute and relative frequencies or averages together with standard deviation (SD) are reported

“Confident user” refers to self-evaluation answers “high” or “very high”, while “Frequent user” is identified when self-reported usage answers were “frequently” or “daily”

§: Statistically significant difference (*p* < .05) by gender;

† Statistically significant difference (*p* < .05) by educational level;

° eHealth user if more than 4 out of 8 proposed experiences were selected;

^ students that identified both tele visits and telemonitoring as telemedicine

*Digital and technology knowledge and skills.* Participants generally displayed positive reactions when learning new computer or smartphone applications, with 21.8% demonstrating strong enthusiasm. Notably, there was no significant difference detected between medical students and resident physicians (WMW *p* = .830; FFH *p* = .874), indicating similar attitudes towards learning new apps within both groups. However, a statistically significant difference in technology enthusiasm based on gender was observed. The WMW test and FFH test (*p* = .001 and *p* = .003, respectively) revealed a significant disparity in responses between male and female participants. Specifically, a higher percentage of females (30.7%) was not keen on learning new apps compared to males (21.1%). Conversely, a higher percentage of males (30.3%) “Really like” learning new apps compared to females (14.2%). The highest levels of ICT proficiency were associated with smartphones, followed by laptops, desktop computers, and tablets. No statistically significant difference in proficiency was detected for each device when testing by student type (desktop, WMW *p* = .212, FFH *p* = .209; laptop, WMW *p* = .905, FFH *p* = .542; smartphone, WMW *p* = .126, FFH *p* = .445; tablet, WMW *p* = .021, FFH *p* = .055). However, gender played a role in perceived ability, with 51.9% of females reporting good levels of proficiency on desktop computers, significantly lower than males (66.9%; WMW *p* < .001; FFH *p* = .001). Conversely, 57.4% of females reported being skilled with laptops, compared to 75.4% in males (WMW *p* < .001; FFH *p* = .004). Regarding the ability to use smartphones (WMW *p* = .263; FFH *p* = .102) and tablets (WMW *p* = .053; FFH *p* = .080), no gender effect was observed. Moving from proficiency to frequency of use, smartphones were used every day by 98.4% of respondents, while daily use of laptops was reported by 61.2%. This frequency decreased to 29.0% and 29.3% for daily interactions with tablets and desktop computers, respectively. The frequency of use showed no detectable gender-specific influence (desktop, WMW *p* = .295, FFH *p* = .032; laptop, WMW *p* = .575, FFH *p* = .700; smartphone, WMW *p* = .226, FFH *p* = .250; tablet, WMW *p* = .594, FFH *p* = .069). However, there were significant differences in utilization rate between the two categories of students for desktop computers (WMW *p* < .001; FFH *p* < .001) and tablets (WMW *p* < .001; FFH *p* < .001). As indicated in Table 2, resident physicians primarily used desktops regularly, whereas medical students used tablets frequently. Regarding the utilization frequency of laptops (WMW *p* = .197; FFH *p* = .315) and smartphones (WMW *p* = .122; FFH *p* = .239), no educational level effect was observed.

In terms of self-evaluation of digital skills, only 2.6% of the sample considered themselves to have basic technology skills. Moderate skills were self-recognized by 12.0%, while the majority perceived their ability as either good (44.3%) or excellent (41.0%). Interestingly, no statistically significant differences (WMW p = .311; FFH p = .279) in terms of digital skills self-evaluation were observed between the types of students. However, a statistically significant difference was found between males and females (WMW p = .005; FFH p = .008) regarding the self-evaluation of digital competencies. When the two higher proficiency levels (“Excellent”, “Good”) and the two lower levels (“Basic”, “Moderate”) were combined, the gender difference became undetectable (FFH p = .628). In fact, the most influential difference between genders was observed within the “Excellent” digital competence category (males, 50.7%; females, 32.7%) and within the “Good” category (males, 35.9%, females 51.2%). The possession of five fundamental digital skills dimensions was investigated through 20 binary items; 82.4% of the sample responded positively to all items. Specifically, Information Retrieval domain was the most frequently selected, followed by Communication, Safety, Problem Solving, and Content Creation. As reported in Table 2, the only two dimensions that were statistically significant across educational levels were Communication (FFH p = .043) and Content Creation (FFH p = .049). No gender effect was observed within the five digital skills proficiency domains.

*Digital Health Knowledge and Skills.* The proportion of respondents having at least one mHealth app on their smartphone did not differ significantly between students and residents (FFH *p* = .793), the percentage of females with such an app (79.6%) was significantly higher compared to their male counterparts (69.0%, FFH *p* = .035). In terms of their personal eHealth experience, irrespective of the mobile tool used, the most frequently reported individual experiences with digital technologies were online information access (98.4%) and obtaining information through articles or videos (98.4%). Checking diagnostic test results was also frequent, with 92.8% of respondents reporting this experience, followed by appointment bookings (80.9%) and receiving updates through medical messaging. Additionally, just over half of the sample (56.7%) had experience in requesting a repeatable medical prescription. The least common usages observed were telemedicine care experiences (17.3%) and participation in medical forums (15.3%).

When comparing these proportions based on academic stage, resident physicians had a statistically significant different proportion from medical students in only two areas of experience: updates on medical issues via messaging (93.6% vs. 70.1%; FFH *p* < .001) and participation in medical forums (20.0% vs. 11.4%; FFH *p* = .040). No significant gender differences were observed in this regard.

**Fig. 1:**
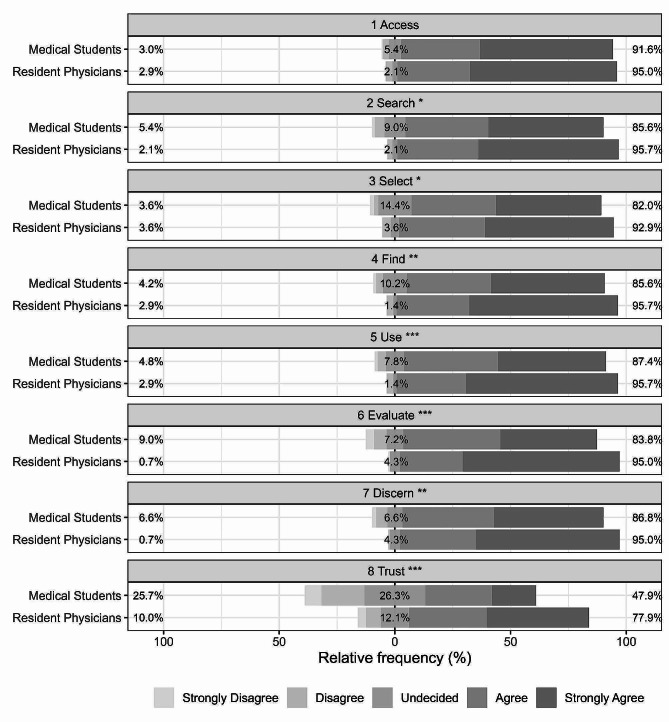
Response rates at items of eHeals scale by educational level. Low, middle and high percentage of agreement are reported for each combination between the eight items and the two educational levels (Medical Students and Resident Physicians). Original items: Access, “I know what health resources are available on the Internet”; Search, ”I know where to find helpful health resources on the Internet”; Select, “I know how to find helpful health resources on the Internet”; Find, ”I know how to use the Internet to answer my questions about health”; Use, “I know how to use the health information I find on the Internet to help me”; Evaluate, “I have the skills I need to evaluate the health resources I find on the Internet”; Discern, “I can tell high quality health resources from low quality health resources on the Internet”; Trust, ”I feel confident in using information from the Internet to make health decisions”. Wilcoxon-Mann-Whitney test p: *, < 0.05; **, < 0.01; ***, < 0.001

Transitioning from the concept of experience to that of skills, the overall distribution of the global IT-eHEALS scale score, designed to assess the level of digital health literacy on a scale ranging from 0 to 32 points, had median value of 28 (interquartile range: 23–32). The maximum score was achieved by 79 (25.7%) participants. In line with the validation conducted by Bravo et al. (2018) [31], the reliability analysis resulted in a Cronbach’s alpha of 0.93 (Feldt’s confidence interval = 0.92–0.94), while the first component was able to explain 70.5% of the total variance (eigenvalue: 5.6). At univariate analyses, medical students had a significantly lower IT-eHEALS score (median, 1st-3rd quartile: 26, 22–31) compared to medical residents (30, 24.75-32; WMW *p* < .001); this difference did not persist between genders (WMW *p* = .562). As additional evidence, in Fig. 1, the single-item results of eHEALS scale are reported by educational level.

Concerning the perception of being informed about telemedicine, only 10.1% of the sample felt not informed at all. The overall trend indicated a low perception of being well-informed: 5.5% considered themselves extremely informed, 37.8% felt sufficiently informed, and 46.6% considered themselves inadequately informed. Resident physicians were significantly more informed than medical students (WMW *p* < .001; FFH *p* < .001), with only 2.1% of residents not considering themselves informed at all, in contrast to 16.8% of students. No statistically significant differences were observed by gender (WMW *p* = .717; FFH *p* = .571).

Furthermore, participants were asked to identify telemedicine tools from a list of remote health management situations. It was found that 82.1% correctly identified teleconsultation, and 87.6% identified telemonitoring. Only 73.0% correctly identified both definitions of telemedicine forms, with no statistically significant differences observed by educational level (FFH *p* = .093) or gender (FFH *p* = .699). However, respondents incorrectly identified the following as telemedicine: accessing examination reports on a PC (65.8%), using chat/WhatsApp to communicate with their primary care physician (48.5%), receiving medical prescriptions via email (63.5%), and booking exams or appointments online (51.1%).

### Behavioural component

As with the technical component, Table 3 summarises the results for the behavioural component of digital readiness.

**Table 3:** Primary summary results of the survey behavioural component, overall and by educational level

Sections	Domains	Overall	Medical Students	Resident Physicians	
B. BEHAVIOURAL COMPONENT					
Previous experience with Telemedicine					
	Heard about	207 (67.4)	97 (58.1)	110 (78.6)	†
	Personal experience	78 (25.4)	32 (19.2)	46 (32.9)	†
	Academic experience	48 (15.6)	11 (6.6)	37 (26.4)	†
	*teleconsultation*	25 (52.1)	5 (45.5)	20 (54.1)	
	*telemonitoring*	19 (39.6)	1 (9.1)	18 (48.6)	†
	*telecontrol*	29 (60.4)	6 (54.5)	23 (62.2)	
	*telerehabilitation*	6 (12.5)	1 (9.1)	5 (13.5)	
Interest in Telemedicine					
	Get training	254 (82.7)	143 (85.6)	111 (79.3)	
	Try usage	248 (80.8)	134 (80.2)	114 (81.4)	
	Future utilization	232 (75.6)	110 (78.6)	122 (73.1)	

Notes: Absolute and relative frequencies are reported

§: Statistically significant difference (*p* < .05) by gender;

† Statistically significant difference (*p* < .05) by educational level;

*Experience with Telemedicine.* A statistically significant difference in familiarity with telemedicine emerged between residents and students (FFH *p* < .001). Specifically, 21.4% of residents had never heard of telemedicine, compared to 41.9% of medical students. This difference became even more pronounced when considering the frequency of exposure to information about telemedicine. In fact, 11.4% of students reported hearing about it more than twice, in contrast to 35.7% of medical residents. No significant disparity based on gender was observed (FFH *p* = .881).

Regarding personal experience, 74.6% of the sample had never been involved in telemedicine, either for themselves or for acquaintances. The remaining portion, for which the frequency of involvement was investigated, can be divided into 42.3% who had encountered telemedicine personally only once, 23.1% once or twice, and 34.6% more frequently. Among those engaged in a personal telemedicine initiative, 59.0% were residents, while 41.0% were students (FFH *p* = .008). There were no statistically significant differences based on gender (FFH *p* = .085). However, in the group of those who had no experience of involvement in personal telemedicine initiatives, a balance between males and females (49.8% and 50.2%, respectively) was observed, compared to 37.7% males and 62.3% females in the group of those involved.

Turning to non-personal involvement during their educational journey, the percentage of those who had never been involved in telemedicine activities rose to 84.4%. The remaining portion, for which the frequency of involvement was investigated, could be divided into 39.6% who had encountered telemedicine personally only once, 14.6% once or twice, and 45.8% more frequently during their educational path. In this case as well, among those engaged in an academic telemedicine initiative, resident physicians were more engaged in their studies (77.1%) compared to medical students (22.9%, FFH *p* < .001). Additionally, no statistically significant differences based on gender were observed (FFH *p* = .058). Nevertheless, a consistent proportion of males and females (49.2% and 50.8%, respectively) remained in the group of those who had no experience of involvement in university telemedicine projects, while in the group of those involved, 2 out of 3 were females. When examining the specific telemedicine services used by those who experienced it during their educational journey, telecontrol and teleconsultation emerged as the most common, followed by telemonitoring and telerehabilitation. Within this subgroup, the only significant difference observed was for telemonitoring based on educational level (FFH *p* = .032), with resident physicians possessing significantly more academic expertise than medical students. No significant gender differences were observed in this regard.

*Interest in Telemedicine.* As reported in Table 3, the importance of receiving training in telemedicine during their studies was perceived by more than four out of five participants, as well as the exploratory use of digital technologies. Conversely, 5.9% of the respondents considered training in telemedicine unnecessary, while 4.9% deemed the experimentation with the use of digital technologies unnecessary. Concerning their future medical practice, three out of four respondents expressed interest in telemedicine, in contrast to the 10.4% who were uninterested. None of these three dimensions displayed statistically significant differences based on gender or educational level.

**Fig. 2:**
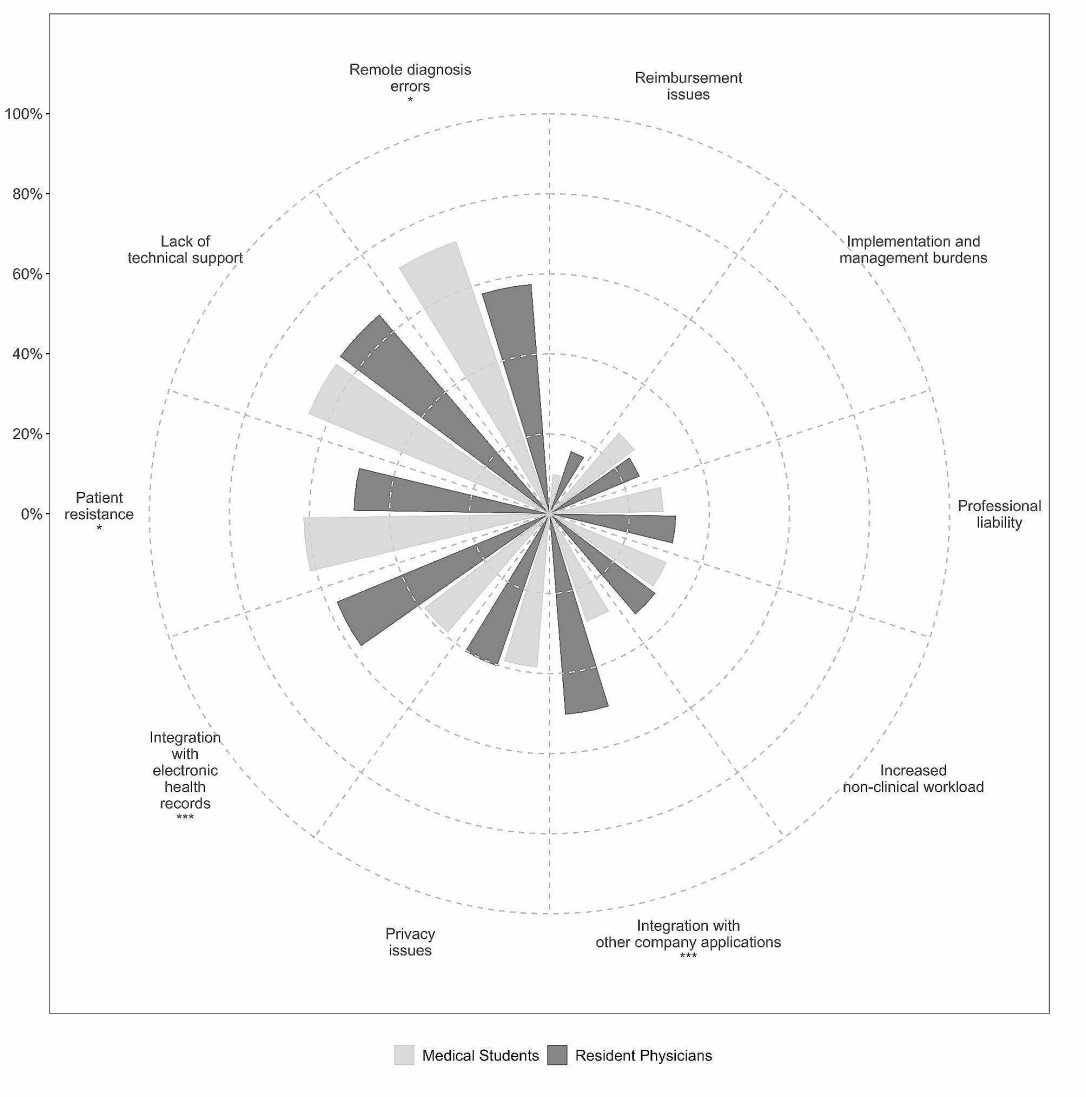
Response rates (%) of telemedicine perceived issues by educational level. The ten investigated dimensions are presented in ascending order of response frequency clockwise. Original question: “From your perspective, what are the current main issues related to Telemedicine?”. Fisher-Freeman-Halton test p: *, < 0.05; ***, < 0.001

*Telemedicine problematic issues.* Participants were asked to identify the main issues associated with telemedicine from a pool of ten options. A discernible heterogeneity emerged in both response frequency and the educational attainment of the subjects. Figure 2 depicts a polar bar plot illustrating the relative frequencies of responses contingent upon the participants’ educational levels.

Starting with the least identified concern, 2.3% of respondents reported having no issues with telemedicine. The reimbursement of telemedicine services appears to be problematic only for 13.0% of participants, followed by the costs related to the platform set-up and maintenance (26.1%), the issues related to professional responsibility (30.3%), and increased effort required in non-clinical activities (32.2%). Concerns about the integration of the telemedicine system with the medical record were selected by 38.3% of medical students, compared to a significantly higher proportion of resident physicians (57.1%, FFH *p* < .001). Privacy issues were reported by 39.7% of participants with no significant difference by educational level. However, the integration of the telemedicine system with other company applications revealed a substantial difference (resident physicians vs. medical students, 57.1% vs. 38.3%, FFH *p* < .001), as well as patient resistance in adopting telemedicine systems (resident physicians vs. medical students, 48.6% vs. 61.1%, FFH *p* = .029). The two most frequently identified issues, each cited by 64% of respondents were lack or inadequacy of technical support and the risk of errors in remotely conducted diagnoses. Specifically, the fear of making diagnostic errors using telemedicine was significantly higher among medical students (70.1%) compared to resident physicians (57.1%, *p* = .023).

Other perceptions, such as the advantages or disadvantages of telemedicine for patients and the advantages for healthcare professionals, were proportionally distributed across the two education levels. Consequently, item-specific results are presented in tabular format in Additional File 2.

## Discussion

The cross-sectional study enabled an examination of the digital readiness of future physicians across two distinct domains: technical and behavioural. Conducted in a country profoundly impacted by the virus, characterized by widespread technology utilization to ensure continuity of care and substantial investment in telemedicine infrastructure, proficiency in technological and digital skills, as well as experience with telemedicine, remains relatively low. However, the findings also revealed a keen interest among both medical residents and students in telemedicine-focused medical education which was not widely available at the time of the survey. This interest suggests a potential to address challenges associated with telemedicine adoption and enhance its acceptance among future healthcare professionals.

The *technical component* results demonstrate that despite post-pandemic enthusiasm for learning new technologies, there still is a lack of technological and digital skills and competences among both medical students and residents. Using validated tools like the eHEALS scale, this study aligns with previous explorative and small-scale studies conducted in European countries [8, 10] and the USA [9, 11], highlighting inadequate digital health training within medical student and resident programs.

There is a generalized perceived difficulty in using ICT devices regardless of the level of medical education. Smartphones were perceived as the easiest to use, whereas desktop computers posed greater challenges. This observation holds particular importance as desktop computers are the predominant technological tools utilized by physicians in Italian hospital settings [36] and they are often outdated. It is relevant to determine whether to integrate the use of smartphones and tablets rather than desktop computers within hospitals, or to increase informatics courses aimed at improving the usability of desktop computers among medical residents and students.

The majority of respondents self-assessed their digital skills as either good or excellent (85%). Nonetheless, gender disparities have surfaced. This raises inquiries regarding the “optimism” expressed by male respondents in contrast to the “underestimation” faced by female respondents. These findings corroborate existing literature on a gender confidence gap in technology usage, wherein women perceive themselves as lacking the same proficiency in ICT as men [37]. This result is also in line with the lower enthusiasm of female respondents in learning to use new technological applications, although women have higher frequency of use of mHealth applications. Women from high-income countries tend to report more eHealth and mHealth usage compared to men [38].

Examining e-health literacy, there was increased familiarity with accessing and using digital health information. However, apprehension persisted in about 38% of cases regarding the application of such information in decision-making. This finding highlights fear and mistrust in proactively using digital health information or health information derived from digital tools, potentially hindering the implementation of digital technologies in medical practices. This outcome aligns with the results of the study conducted to validate the Italian version of the eHEALS [31].

Moving to the *behavioural component*, a significant percentage of participants (75%) lacked direct experience with telemedicine, with medical residents showing greater familiarity than medical students. To the best of our knowledge, this is the first study to address this gap according to the level of medical education. As the University of Milan doesn’t offer specific formal telemedicine curricula, the experience with telemedicine stems from personal care pathways or professional activities during internship, which are increasingly prevalent among residents. Consistently, the knowledge of telemedicine also increased with the increase in medical education levels. While 42% of students admitted to never having heard about telemedicine, 79% of residents acknowledged their awareness. Accordingly, results confirm the limited exposure to telemedicine within traditional medical courses, with only 16% of students and residents reporting any educational interaction with telemedicine.

Consistent with previous research [8–11] most future physicians showed a strong interest in receiving training in telemedicine and incorporating telemedicine into their future clinical practice with no significant distinctions based on gender or education level. In comparison to the pre-pandemic period [11], enthusiasm for utilizing telemedicine and the desire for relevant education have grown, even among those without prior experience. The study’s results highlight the need for formal telemedicine courses in medical education, along with the identification of problematic issues related to the adoption of telemedicine.

The incorporation of formal telemedicine courses into medical education necessitates a comprehensive approach to empower students and residents to become effective physicians. Primarily, it is imperative to ensure that students and residents acquire the requisite technological skills and knowledge [39], building confidence and trust in technological platforms [40], thus promoting the proactive use of digital health information.

The integration of multiple digital platforms and applications, along with the need for technical support, constitutes a major concern among residents. This concern may stem from practical situations they have encountered in telemedicine real-life experience. The telemedicine curriculum should therefore offer an overview of different telemedicine tools and platforms. This should equip students since the first years of medical education with essential competencies for effective virtual patient care and enhance their ability to address challenges in professional settings. The course must also address ancillary themes such as patient privacy and data security [41], which are crucial for maintaining confidentiality and trust in a digital healthcare environment.

Additionally, according to previous studies [42], a relational concern was identified. In particular, medical students expressed apprehension regarding patient resistance (61%). Therefore, telemedicine courses should emphasise the understanding of the various dynamics of patient relationships in telemedicine, recognizing potential drawbacks such as the absence of physical interaction and its influence on patient rapport.

Connected to this issue, medical students indicated a high level of concern about legal considerations, such as potential diagnostic errors (70%). Residents, who have more experience with telemedicine, feel less concerned about this issue (57%). These considerations align with existing literature, which suggests that exposure to telemedicine platforms also facilitates an understanding of the complex ethical, legal, and regulatory challenges associated with this technology. Medical professionals must not only acquire technical skills but also learn to utilize telemedicine in a professional, safe, and evidence-based manner [43]. To address these issues, it is imperative that telemedicine education combines theoretical teaching with practical experience (through simulations) [11, 12], enabling medical students to engage in real-world applications during their clinical rotations and residencies.

Ensuring that clinical settings for internships and residencies are equipped with telemedicine platforms will better prepare future physicians for the evolving healthcare landscape.

This study acknowledges potential limitations, including the questionnaire’s size, which might have influenced participation and response quality. Additionally, the data collection method and uncontrolled variables could have introduced biases, necessitating cautious interpretation of the results.

## Conclusion

It is irrefutable that, in recent years, healthcare has undergone profound changes driven by technological advancements, particularly in telemedicine [44, 45]. This study highlights the need for healthcare workers, including medical students and residents, to receive specific digital health and telemedicine education. The call for introducing curricula and courses in this domain is essential for addressing the challenges of the digital healthcare era and aligning with healthcare visions and missions [46].

This study represents the inaugural exploration of medical students’ and residents’ readiness for the post-pandemic digital health era. By employing a dual-component approach encompassing both technical and behavioural aspects, it contributes valuable insights to the ongoing discourse on digital health and telemedicine training.

Several promising avenues for future research emerge from this study. Firstly, expanding investigations nationally and internationally is essential. While this study focuses on a major university in Northern Italy with a proactive stance on telehealth after the COVID-19 pandemic, understanding how these dynamics extend to a broader context involving diverse educational institutions and international settings is crucial. Examining regional disparities and cultural variations will offer a more comprehensive understanding of training needs and attitudes toward telemedicine and digital health.

Secondly, constructing models will be pivotal to verifying relationships between different dimensions. Given the cross-sectional nature of this study, establishing causal relationships is challenging. Future research could delve into how the technical dimension, encompassing skills and knowledge, influences the behavioural dimension, including attitudes and intentions toward telemedicine. Developing models will shed light on how acquiring technical skills impacts the acceptance and adoption of telemedicine among medical students and healthcare professionals and is pivotal for designing targeted training initiatives.

Understanding how subjective factors, such as self-efficacy and individual interest in telemedicine interact with technical competence will be instrumental in determining the willingness of individuals to adopt this evolving healthcare delivery mode. Constructing models that scrutinize these connections will pinpoint key factors influencing the decisions and conduct of both medical students and healthcare professionals.

Moreover, the validation of future scale scores becomes paramount to enhance the precision of variable measurements. Developing specific tools and indicators tailored to accurately capture digital skills, attitudes, experience, and behavioural intentions is crucial. This validation process ensures the reliability and comparability of data, fortifying the empirical foundation for future research and guaranteeing that the tools employed are apt for assessing the dimensions under scrutiny.

All the proposed advancements within this study are imperative for developing more effective educational programs. By preparing medical students and healthcare professionals to confront the challenges of the digital era, these initiatives aim to foster a workforce that is well-equipped to navigate and excel in the evolving landscape of healthcare.

### Electronic supplementary material

Below is the link to the electronic supplementary material.

Supplementary Material 1

Supplementary Material 2

## Data Availability

The datasets used and analysed during the current study are available from the corresponding author on reasonable request.

## Abbreviations

FFH: Fisher-Freeman-Halton
ICT: Information and Communication Technology
NRRP: National Recovery and Resilience Plan
PC: Personal computer
WMW: Wilcoxon-Mann-Whitney
