# Supplementary material for: The digital readiness of future physicians: nurturing the post-pandemic medical education
Source: BMC Health Serv Res. 2024 Aug 2;24:885. doi: 10.1186/s12913-024-11365-6 (PMC11297791; doi:10.1186/s12913-024-11365-6)
Supplement: Supplementary file 1 — Supplementary Material 1 [file 12913_2024_11365_MOESM1_ESM.pdf]

Personal details

* 1. Gender:
- ◯ Male
- ◯ Female
- ◯ I prefer not to specify

* 2. Age
[   ] years

* 3. Please indicate in which course of study you are:
- ◯ Specialised school
- ◯ Medical student

* 4. What year of study are you attending? Please indicate a number:
[   ] year

Digital and technology knowledge and skills

* 5. What is your reaction when you need to learn to use new computer programs or apps or Smartphones?
- ◯ Detext
- ◯ I am not particularly enthusiastic
- ◯ I find it interesting
- ◯ I like it

* 6. What types of information and communication technology (ICT) devices do you have at home?
(please also select multiple options)
- ☐ Desktop/fixed computer
- ☐ Laptop
- ☐ Smartphone device
- ☐ Tablet device
- ☐ Other ICT devices

7. How do you rate your level of knowledge when using a computer/laptop/tablet/other digital device?

	Very low	Low	Average	Tall	Very High
*Desktop/fixed computer	◯	◯	◯	◯	◯
*Laptop	◯	◯	◯	◯	◯
*Smartphone	◯	◯	◯	◯	◯
*Tablets	◯	◯	◯	◯	◯
*Other	◯	◯	◯	◯	◯

8. Do you use any of the following technologies in your daily routine? If yes, please also indicate the frequency of use.

	Every day	Often	Once a week	Once a month	Sometimes	Never
*Desktop/fixed computer	◯	◯	◯	◯	◯	◯
*Laptop	◯	◯	◯	◯	◯	◯
*Smartphone	◯	◯	◯	◯	◯	◯
*Tablets	◯	◯	◯	◯	◯	◯
*Other	◯	◯	◯	◯	◯	◯

* 9. Do you have access to the internet and wireless connection at home?
- ◯ Yes
- ◯ No

10. Please indicate which of the following digital skills you have

INFORMATION

	Yes	No
*They are able to search for information online using a search engine.	◯	◯
*I know that not all information online is reliable.	◯	◯
*They are able to save or store files or content (e.g. text, images, music, videos, web pages) and retrieve them once they are saved or stored.	◯	◯

COMMUNICATION

	Yes	No
*I am able to communicate with others using your mobile phone, Voice over IP (e.g. Skype), e-mail or chat – using basic functions (e.g. voice messaging, SMS, sending and receiving emails, text exchange).	◯	◯
*I am able to share files and content using simple tools	◯	◯
*I know that I can use digital technologies to interact with services (such as public administrations, banks, hospitals).	◯	◯
*I know social networking sites and online collaboration tools.	◯	◯
*I am aware that some communication rules need to be applied when using digital tools (e.g. when commenting on posts, sharing personal information, etc.)	◯	◯

CONTENT CREATION

	Yes	No
*They are able to produce simple digital content (e.g. text, tables, images, audio files) in at least one format using digital tools.	◯	◯
*They are able to make basic changes to content produced by others.	◯	◯
*I know that content can be covered by copyright	◯	◯
*They are able to apply and modify simple functions and settings of software and applications they use (e.g. change default settings)	◯	◯

SECURITY

	Yes	No
*I am able to take basic measures to protect my devices (e.g. by using antivirus and password)	◯	◯
*I am aware that my credentials (username and password) can be stolen.	◯	◯
*I know that I should not disclose private information online	◯	◯
*I know that excessive use of digital technology can affect my health.	◯	◯

PROBLEM SOLVING

	Yes	No
*They are able to find support and assistance when a technical problem occurs or when using a new device, programme or application.	◯	◯
*I know how to solve some routine problems (e.g. to close a program, restart your computer, reinstall/update a program, check your internet connection).	◯	◯
*I know that digital tools can help me solve problems. I am also aware that they have limitations.	◯	◯
*When I face a technological or non-technological problem, I am able to use the digital tools I know to solve it.	◯	◯

* 11. How do you assess your competence in using digital technologies? (Computer; Smartphones; Tablet; Internet; Desktop applications e.g. Office package; Social media, etc.):

◯ Base
◯ Moderate
◯ Good
◯ Very good

Digital health knowledge and skills

* 12. Does your mobile phone have applications that help you manage or monitor your health status (e.g. applications of health maintenance organisations, testing, various health related parameters)?

◯ Yes
◯ No
◯ Do not know
◯ I don't have a smartphone

* 13. Using technology, she never received...:
(please also select multiple options)

☐ Read an article or watch a video on medical topics.

- [ ] Receive updates on medical issues by email/text message
- [ ] Consult or read information on medical illnesses, medicines or treatments.
- [ ] Monitoring the results of diagnostic tests
- [ ] Book an appointment from your doctor
- [ ] Request a repeatable medical prescription
- [ ] Take part in an online treatment session with a doctor/nurse/dietician etc.
- [ ] Take part in medical forums for consultation

14. With regard to internet usage to search for information about your health or that of a family member, please indicate your degree of agreement with the following statements:

	Totally disagree	Disagree	Nor do you disagree.<br>I do not agree	Enough agree	Very agree
*I know how to find useful health information on the internet	○	○	○	○	○
*I know how to use the Internet to answer health questions	○	○	○	○	○
*I know what health information is available on the Internet	○	○	○	○	○
*I know where to find useful health information on the internet	○	○	○	○	○
*I know how to use the health information I find on the internet so that it can help me	○	○	○	○	○
*I have the skills I need to assess the health information I find on the internet	○	○	○	○	○
*I know the low or high quality of health information I find on the internet	○	○	○	○	○
*I feel confident using the information I find on the internet to make decisions about my health	○	○	○	○	○
*I started using the internet to find information about his health only after Covid	○	○	○	○	○
*I consulted the internet to find information on the COVID-19 vaccine	○	○	○	○	○

* 15. How well do you feel informed about telemedicine?

- ○ Very well informed
- ○ Sufficiently informed
- ○ Poorly informed
- ○ Not at all informed

* 16: In your opinion, which of the following situations would it fall within the definition of Telemedicine?
(please also select multiple options)

- [ ] Remote viewing via video call
- [ ] Send your health data in real time to your doctor or hospital (pressure, cardiogram, glycaemia...)
- [ ] Use chat or whatsapp to communicate with your doctor
- [ ] Receive medical prescription via email
- [ ] See test reports carried out directly by the computer
- [ ] Be able to book online examinations or visits
- [ ] None of these

Previous experience with Telemedicine

* 17. During your course of study, have you ever heard of telemedicine?

- ○ No, never
- ○ Yes, once/twice
- ○ Yes, more than twice

* 18. Have you already been involved in telemedicine projects for you or someone you know?

- ○ No, never
- ○ Yes, once
- ○ Yes, two/three times
- ○ Yes, more than three times

* 19. Have you already been involved in telemedicine projects during your course of study or traineeship/specialisation?

- ○ No, never
- ○ Yes, once
- ○ Yes, two/three times
- ○ Yes, more than three times

20. If you have been involved in telemedicine experiences during your course of study or traineeship/specialisation, which of the following services have you used?

	Yes	No
*Tele-visit<br>in real time between doctor and patient using computers, tablets or smartphones, with the possibility to exchange clinical data, reports or images if necessary.	◯	◯
*Tele-monitoring remote<br>detection and transmission via sensors and devices, on a continuous basis, of vital and clinical parameters to the doctor or hospital for monitoring the evolution of the disease or patient's treatment at home.	◯	◯
*Tele-medical check-up<br>series of contacts by the doctor, by means of calls or video calls, including the sharing of clinical data collected by the patient, to monitor the progress of the patient's clinical picture,	◯	◯
*Remote<br>rehabilitation tele-rehabilitation in real time using computers, tablets or smartphones, with the possibility to share clinical data, reports or images if necessary.	◯	◯

Interest in Telemedicine

21. Please express your degree of agreement with the following sentences

	Totally disagree	Little agree	Nor do you disagree. I do not agree	Rather agree	Strongly agree
*I believe that receiving telemedicine training during your studies is important	◯	◯	◯	◯	◯
*I believe that experimenting with the use of digital technologies (such as telemedicine) during your studies is important	◯	◯	◯	◯	◯
*I would be interested in using telemedicine in my future practice	◯	◯	◯	◯	◯

* 22. In your opinion, what are the general **benefits** of Telemedicine for patients?
(please also select multiple options)

- ☐ The patient saves the time it takes to reach the hospital/clinic
- ☐ Patient saves waiting time in an outpatient
- ☐ Save money for the patient (travel, absence from work, etc.)
- ☐ Avoids the need to be accompanied by visits
- ☐ Allows you to use healthcare facilities away from home
- ☐ Makes it easier to explain your symptoms
- ☐ Makes it easier to contact your doctor/healthcare professional
- ☐ Allows for more frequent checks/visits/sessions
- ☐ Reduces health costs
- ☐ Makes patients more comfortable
- ☐ Facilitate booking
- ☐ Allows the doctor/healthcare professional to have more data for the treatment of the patient
- ☐ Allows you to talk to multiple doctors/health professionals at the same time
- ☐ Other advantages: ___
- ☐ Has no advantage

* 23. What do you think are the main **disadvantages** of Telemedicine for patients?
(please also select multiple options)

- ☐ Limits the possibility to ask questions to the health professional
- ☐ Limits personal relationship with the health professional
- ☐ It is not as effective as a real visit to make a diagnosis or control
- ☐ Increased time to get a visit
- ☐ It is subject to technical communication problems (e.g. connection disruptions, poor image quality...)
- ☐ Requires the patient's ability to use the technology
- ☐ Requires the patient to possess specific technological tools
- ☐ Patient needs support and explanations to learn how to use it
- ☐ Is subject to privacy problems
- ☐ Health costs increase
- ☐ Creates a division between the most used and less-favoured patients
- ☐ Challenges older patients
- ☐ Other disadvantages: ___
- ☐ There is no disadvantage

* 24. From your point of view, what are the main issues related to Telemedicine at present?
(please also select multiple options)

- ☐ Privacy issues (e.g. risk of data theft, etc.)
- ☐ Issues related to professional responsibility
- ☐ Integration of the telemedicine system with the medical record
- ☐ Integration of the telemedicine system with other company applications
- ☐ The increased effort required in non-clinical activities (e.g. time for technology management)
- ☐ Lack or inadequate technical support
- ☐ Increased costs related to the implementation and operation of telemedicine platforms
- ☐ Patient resistance to telemedicine systems
- ☐ Issues related to the reimbursement of benefits

- [ ] Risk of errors in remote diagnosis
- [ ] Other issues: ___
- [ ] There is no problem

* 25. What do you think are the main benefits in Telemedicine for health professionals?
(please also select multiple options)

- [ ] Allows for simplification of the bureaucratic part of the work
- [ ] Allows you to work more in an optimised way
- [ ] Allows you to devote more time to the patient
- [ ] Allows for more flexibility in the management of working time
- [ ] Allows for greater diagnostic accuracy
- [ ] Allows for a higher quality of the clinical data collected
- [ ] Allows you to work better in teams
- [ ] Enables real collaboration between local medicine and specialists
- [ ] Issues related to the reimbursement of benefits
- [ ] Other benefits for the trader: ___
- [ ] No benefit for the trader

Please verify that you are human

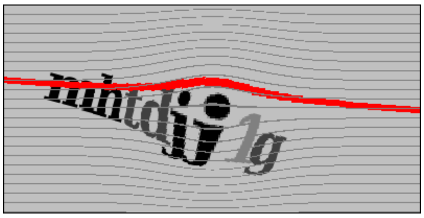

Please enter the displayed text

Submit
